# Genetic Evidence Linking Inflammatory Cytokines and Blood Metabolites to Heart Failure Risk

**DOI:** 10.1155/cdr/6340671

**Published:** 2026-05-11

**Authors:** Yanbing Yao, Linghui Tang, Peilin Zhou, Feng Huang, Zhiyu Zeng

**Affiliations:** ^1^ Department of Cardiology, The First Affiliated Hospital of Guangxi Medical University, Nanning, Guangxi, China, gxmu.edu.cn; ^2^ Guangxi Key Laboratory of Precision Medicine in Cardio-Cerebrovascular Diseases Control and Prevention, Nanning, Guangxi, China, gxmu.edu.cn; ^3^ Guangxi Clinical Research Center for Cardio-Cerebrovascular Diseases, Nanning, Guangxi, China, gxmu.edu.cn; ^4^ School of Nursing, Guangxi Medical University, Nanning, Guangxi, China, gxmu.edu.cn; ^5^ Department of Bone and Joint Surgery (Guangxi Diabetic Foot Salvage Engineering Research Center/Research Centre for Regenerative Medicine), The First Affiliated Hospital of Guangxi Medical University, Nanning, Guangxi, China, gxmu.edu.cn

**Keywords:** heart failure, inflammatory cytokines, Mendelian randomization, metabolites, uPA

## Abstract

**Background:**

Heart failure (HF) is a critical condition characterized by the heart′s inability to pump blood effectively, leading to significant morbidity and mortality. Inflammation and metabolic disturbances play key roles in its progression. This study is aimed at elucidating the causal relationships between inflammatory cytokines, metabolites, and HF using Mendelian randomization (MR).

**Methods:**

A three‐step, two‐sample MR analysis was performed using genetic data from genome‐wide association studies. The study included data on 91 inflammatory cytokines, over 1400 metabolites, and HF cases from the FinnGen project, comprising 29,672 cases and 382,509 controls. Single‐nucleotide polymorphisms with significant associations (*p* < 1 × 10^−5^) were selected as instrumental variables. The inverse variance–weighted method was the primary analytical tool, supplemented by MR‐Egger, weighted median, simple mode, and weighted mode methods. Heterogeneity was assessed with Cochran′s *Q* test, and pleiotropy was evaluated using MR‐Egger intercept and MR‐PRESSO. Robustness was confirmed through leave‐one‐out sensitivity analysis.

**Results:**

The MR analysis identified eight inflammatory cytokines significantly associated with HF risk. Elevated levels of three cytokines (FGF19, MMP‐1, and TNF‐*β*) were linked to an increased risk of HF, whereas five cytokines (DNER, IL‐10, uPA, TNFSF12, and LIFR) were linked to a decreased risk of HF. Additionally, 12 metabolites were found to be significantly associated with HF, with 6 (2PY, BCG, NAG/NAGal, X‐24801, X‐21283, and X‐22776) enhancing risk and 6 (PC, 3HL, 2‐BG, X‐23659, X‐25422, and X‐24546) showing protective effects. Notably, mediation analysis indicated that certain metabolites, X‐22776, mediated the effect of uPA on HF.

**Conclusions:**

This study provides novel genetic evidence supporting the causal role of inflammatory cytokines in HF and highlights the mediating role of metabolites. These findings offer new insights into the pathogenesis of HF and suggest potential biomarkers and therapeutic targets for its prevention and treatment.

## 1. Introduction

Heart failure (HF) is a complex clinical syndrome characterized by the heart′s inability to pump sufficient blood to meet the body′s metabolic demands, leading to symptoms such as shortness of breath, fatigue, and fluid retention [1]. HF is a major global health problem, with an estimated prevalence of over 64 million people worldwide, and its incidence is increasing due to an aging population and improved survival from other cardiovascular diseases [2]. The pathophysiology of HF is multifactorial, involving a complex interplay of neurohormonal, hemodynamic, and inflammatory pathways [3]. Inflammation has been recognized as a key contributor to the progression of HF, with elevated levels of inflammatory cytokines such as tumor necrosis factor‐alpha (TNF‐*α*), interleukin‐6 (IL‐6), and interleukin‐1*β* (IL‐1*β*) observed in patients with HF [4–6]. These cytokines are believed to promote adverse cardiac remodeling and contribute to the deterioration of cardiac function [7]. Despite advances in the management of HF, including pharmacological treatments such as beta‐blockers, ACE inhibitors, and aldosterone antagonists, as well as device‐based therapies, mortality and morbidity remain high [8].

Recent research has highlighted the potential role of metabolic dysregulation in the development and progression of HF [9, 10]. Metabolites, the end products of cellular processes, reflect the body′s metabolic state and have been linked to various pathophysiological processes in HF, including oxidative stress, mitochondrial dysfunction, and altered substrate utilization [11]. Disturbances in lipid metabolism, such as increased circulating free fatty acids and decreased high‐density lipoprotein levels, have been associated with worse outcomes in HF patients [12]. Additionally, accumulating evidence suggests that metabolic derangements can interact with inflammatory pathways, potentially creating a vicious cycle that exacerbates HF progression [13, 14]. Inflammatory cytokines may alter metabolic pathways, leading to the accumulation of harmful metabolites that further drive inflammation and cardiac dysfunction [6]. However, the precise causal relationships between inflammatory cytokines, metabolites, and HF remain unclear.

Mendelian randomization (MR) has emerged as a powerful tool to investigate causal relationships in epidemiology by using genetic variants as instrumental variables (IVs) to infer the effect of an exposure on an outcome, thus reducing the risk of confounding and reverse causation [15]. MR studies have been successfully applied to elucidate the causal roles of inflammatory cytokines and metabolic traits in various diseases, including cardiovascular diseases [16]. Recent MR studies have investigated the causal effects of inflammatory cytokines on HF, but were limited to a narrow range of cytokines and did not explore the mediating role of metabolites [17]. To address these gaps, the present study comprehensively analyzed 91 inflammatory cytokines and over 1400 metabolites, and further employed mediation MR analysis to explore whether metabolites mediate the inflammatory cytokine–HF relationship. The first step involves assessing the causal effects of a comprehensive panel of inflammatory cytokines on HF risk. The second step involves assessing the causal effects of metabolites on HF risk. The third step evaluates the potential mediation of these effects by specific metabolites, providing insights into the complex interplay between inflammation, metabolism, and HF. This approach has the potential to identify novel biomarkers and therapeutic targets for HF, particularly those related to lipid metabolism and inflammatory pathways.

## 2. Methods

### 2.1. Data Collection

For this investigation, we utilized plasma inflammatory cytokines information derived from a comprehensive proteomic quantitative trait locus (pQTL) analysis published in 2023 [18]. The aforementioned study employed the Olink Target inflammation panel to analyze 91 inflammation‐associated plasma proteins from 14,824 participants of European ancestry. The genome‐wide association study (GWAS) summary statistics for these inflammatory mediators are publicly accessible via the GWAS Catalog under accession numbers ranging from GCST90274758 to GCST90274848 (https://www.ebi.ac.uk/gwas/home).

Genetic information for plasma metabolites was obtained from a GWAS conducted within the Canadian Longitudinal Study on Aging, released in 2023, encompassing data from 8299 individuals of European descent [19]. A total of 1091 blood metabolites were quantified using the Metabolon HD4 platform with batch normalization applied to maintain consistency, alongside 309 metabolite ratios. The GWAS summary data for these metabolic compounds are publicly available through the GWAS Catalog under Accession Numbers GCST90199621 through GCST90201020.

Data concerning HF were sourced from the FinnGen Consortium, where cases were identified using the ICD‐10 Code I50 (HF, including all subtypes). This consortium represents a large‐scale biomedical research initiative established through collaboration among multiple Finnish research institutions and hospitals, designed to investigate gene–disease relationships by integrating genetic information with electronic health records. The project conducts comprehensive genomic analyses of the Finnish population to examine the genetic foundations of diseases, risk factors, and their interactions with environmental elements. The consortium provides integrated datasets that combine genetic profiles with electronic medical records. Our analysis employed data from the FinnGen Consortium′s 10th release (R10) published in 2023, which contains 29,672 cases of HF and 382,509 control subjects. The corresponding GWAS summary statistics are accessible through FinnGen′s public repository (gs://finngen‐public‐data‐r10/summary_stats/finngen_R10_I9_HEARTFAIL.gz).

### 2.2. Study Design

**Figure 1:**
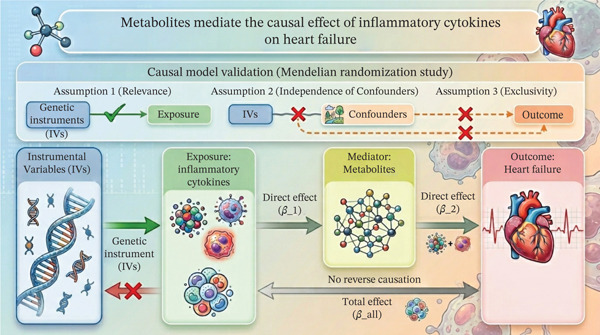
The study design. A three‐step MR study of inflammatory cytokines on HF mediated by metabolites.

Different from previous two‐sample MR analyses, a three‐step MR analysis was employed to assess the causal relationships between inflammatory cytokines, metabolites, and HF (Figure 1) [[20, 21]. The first step used the inverse variance–weighted (IVW) method as the primary analytical approach to estimate the total effect of inflammatory cytokines and metabolites on HF. To ensure robustness and account for potential pleiotropy, additional MR methods such as MR‐Egger, weighted median, simple mode, and weighted mode were applied [22, 23]. In the second step, we performed MR analyses to estimate the causal effects of inflammatory cytokines on specific metabolites. The third step involved investigating the pathways through which cytokines may influence HF via metabolites. To assess the mediation role of metabolites, we examined the causal effect of inflammatory cytokines on the selected metabolites associated with HF risk. The indirect effect was then estimated using the “product of coefficients” method, and the *δ* method was used to calculate the standard error of these indirect effects.

### 2.3. Genetic IVs Selection

Single‐nucleotide polymorphisms (SNPs) were selected as IVs based on their associations with inflammatory cytokines, metabolites, and HF. In order to identify a sufficient number of SNPs, a uniform *p* value threshold of less than 1 × 10^−5^ was applied across all three exposure categories to select candidate IVs. Genetic variants were clumped using an *R* ^2^ threshold of less than 0.001 and a clumping distance of 10,000 kb to ensure independence. The *F*‐statistic was calculated as the ratio of the effect size (*β*) to the square of the standard error, with a cut‐off value of 10, to ensure the strength of the IVs. Potential confounders, including age, sex, ethnicity, and comorbid conditions, were identified using the PhenoScanner V2 database (http://www.phenoscanner.medschl.cam.ac.uk/).

### 2.4. Statistical Analysis

We conducted MR analysis using R software (Version 4.3.2) and its “TwoSampleMR” and “gwasglue” packages to explore the causal relationships between 91 plasma inflammatory cytokines and 1400 metabolites with HF risk. Effect sizes were expressed as odds ratios (OR) and 95% confidence intervals (CI), calculated using the formulas OR = exp (*β*_IVW) and 95% CI = exp (*β*_IVW ± 1.96 × SE_IVW), where *β*_IVW was obtained through the weighted average of individual SNP effects: *β*_IVW = *Σ* (*β*_YX × *w*)/*Σ* *w*, with weights (*w*) based on the inverse of the standard errors of the SNP‐outcome associations.

To ensure the robustness of our findings, we implemented a series of sensitivity analyses, with specific details referenced in the exclusion restriction assumption section under instrumental variable selection. To evaluate the consistency and robustness of the MR findings, heterogeneity was assessed using Cochran′s *Q* test (significance threshold: *p* > 0.05). The MR‐Egger intercept test was employed to detect pleiotropy (significance threshold: *p* > 0.05), whereas the MR‐PRESSO global test and outlier removal procedures were applied when significant pleiotropy was detected [24]. Additionally, leave‐one‐out analysis was performed to explore the influence of potential outlying genetic variants on the overall estimates.

The Mendelian mediation analysis calculated the influence of mediating variables through three key regression equations: first measuring the total effect of different inflammatory cytokines on HF (*β*_all), then measuring the effect of different metabolites on HF (*β*_2_) and the effect of inflammatory factors on metabolites (*β*_1_), and finally calculating the indirect effect (*β*_1_ × *β*_2_) and direct effect (*β*_all—*β*_1 × *β*_2). The mediation analysis was performed using the MendelianRandomization software package, with the mediation proportion calculated using the formula: Mediation proportion = (*β*_1_ × *β*_2_)/*β*_all. The significance of mediation effects was validated through statistical techniques such as the Sobel test or bootstrap methods, where a significantly nonzero indirect effect indicated the presence of a mediation effect.

## 3. Results

### 3.1. MR Analysis of Inflammatory Cytokines on HF Risk

**Figure 2:**
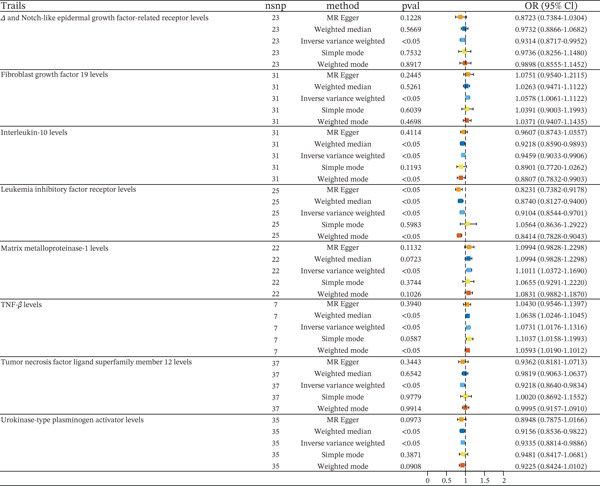
MR analysis of inflammatory cytokines and HF risk.

The study selected 31,908, 2,725 and 81 SNPs as IVs for 1400 metabolites, 91 inflammatory cytokines, and HF (Tables S1–S3). In the analysis of inflammatory cytokines using the five methods, eight inflammatory cytokines demonstrated statistically significant associations (Figure 2). In IVW method, fibroblast growth Factor 19 (FGF19) had an OR of 1.0578 (95% CI: 1.0061–1.1122), matrix metalloproteinase‐1 (MMP‐1) had an OR of 1.1011 (95% CI: 1.0372–1.1690), and TNF‐beta (TNF‐*β*) had an OR of 1.0731 (95% CI: 1.0176–1.1316). In weighted median and weighted mode method, TNF‐*β* had an OR of 1.0638 (95% CI: 1.0246–1.1045) and an OR of 1.0593 (95% CI: 1.0190–1.1012). All these findings indicate potential roles in inflammation processes with statistically significant results and *p* < 0.05.

On the other hand, in IVW method, Delta and Notch‐like epidermal growth factor‐related receptor (DNER) had an OR of 0.9314 (95% CI: 0.8717–0.9952), interleukin‐10 (IL‐10) had an OR of 0.9459 (95% CI: 0.9033–0.9906), urokinase‐type plasminogen activator (uPA) had an OR of 0.9335 (95% CI: 0.8814–0.9886), and tumor necrosis factor ligand superfamily Member 12 (TNFSF12) had an OR of 0.9218 (95% CI: 0.8640–0.9834), with leukemia inhibitory factor receptor (LIFR) showing an OR of 0.9104 (95% CI: 0.8544–0.9701). In weighed median method, IL‐10 had an OR of 0.9218 (95% CI: 0.8590–0.9893), LIFR had an OR of 0.8740 (95% CI: 0.8127–0.9400), and uPA had an OR of 0.9156 (95% CI: 0.8536–0.9822). In MR‐Egger method, LIFR had an OR of 0.8231 (95% CI: 0.7382–0.9178). These results suggest a decreased risk of the associated conditions when levels of these inflammatory factors are higher, emphasizing the complex interactions between different cytokines and their potential impact in pathological states.

We conducted a series of sensitivity analyses to assess the robustness of the results and explore potential biases and the validity of the IVs. First, we used the multiple IVs method to examine the potential bias introduced by a single instrument. Additionally, to further evaluate the validity of the instrumental variables, we performed a heterogeneity test using Cochran′s *Q* statistic. The results showed that the *Q* statistics for all instrumental variables were not significant (*p* > 0.05), indicating the absence of substantial heterogeneity (Table S4). Therefore, we conclude that the effects of the selected IVs are consistent across different study samples and adequately represent variations in the exposure factor. We also conducted a directional hypothesis test using Egger regression and PRESSO to examine potential reverse causality and instrument bias. Both Egger regression and PRESSO results did not show significant bias, suggesting that our MR findings are unlikely to be influenced by bias (Table S5). To further validate our conclusions, we performed a leave‐one‐out analysis, systematically excluding each IV and rerunning the MR analysis. The results showed that the estimated causal effect remained relatively stable, with only minor variations when any single instrument was removed, indicating that our primary findings are robust (Figure S1). Overall, the results of the sensitivity analyses support the robustness of the MR analysis, ruling out common biases and confounding factors, and strengthening the credibility of the causal relationship between the exposure factor and the outcome.

**Figure 3:**
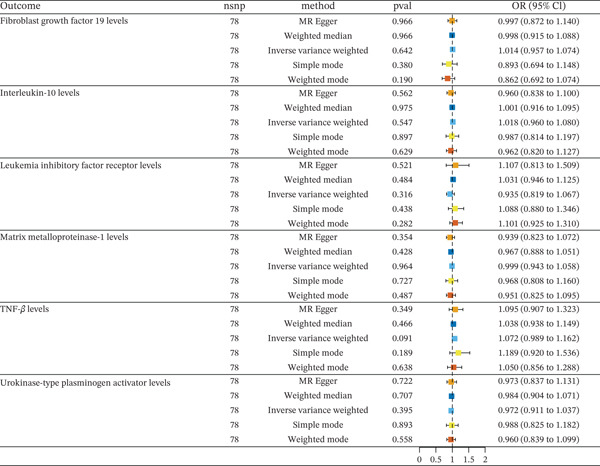
Reverse MR analysis of HF risk on inflammatory cytokines.

Then, we conducted a reverse MR analysis of these eight inflammatory cytokines on HF. The results of the reverse MR analysis showed HF can reverse act on TNFSF12 and DNER (Table S6). So next, we will explore the impact of the remaining six inflammatory factors on HF via metabolites (Figure 3). The results of the sensitivity analyses also support the robustness of the MR analysis (Table S7 and Figure S2).

### 3.2. MR Analysis of Metabolites on HF Risk

**Figure 4:**
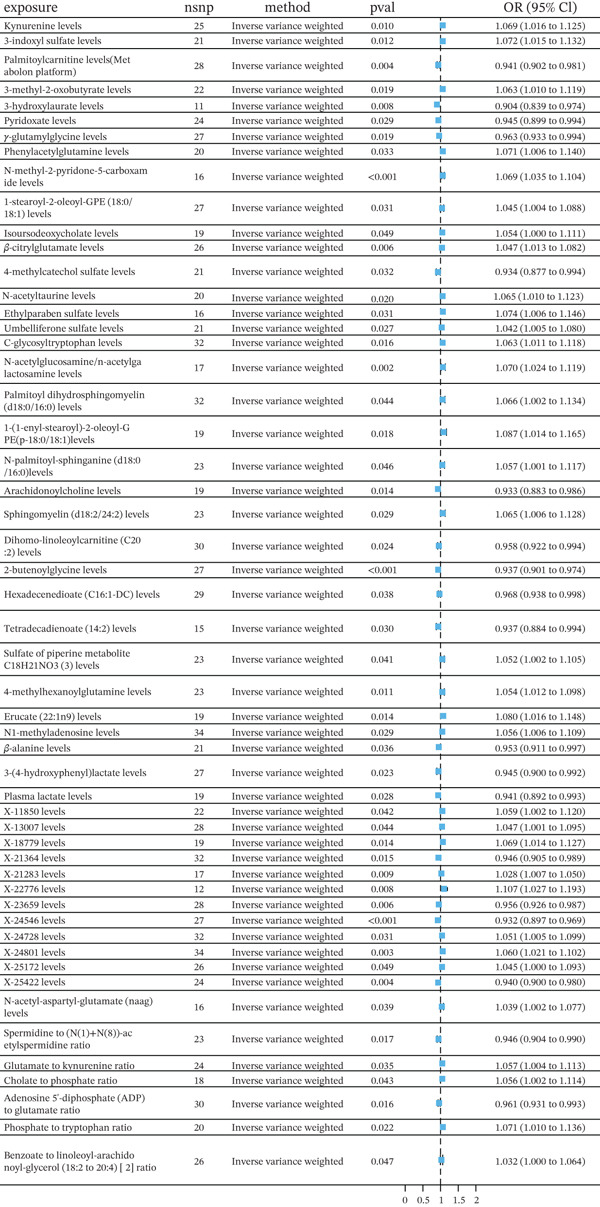
Forest plots showing the association of various metabolites with HF risk, analyzed using the IVW method.

We first identified 53 metabolites that were associated with HF risk in Figure 4. Then, to narrow the scope, we set *p* < 0.01 and identified 12 metabolites significantly associated with HF risk (Table S8).

Six metabolites were associated with an increased risk of HF, as indicated by an OR greater than 1. N‐methyl‐2‐pyridone‐5‐carboxamide (2PY) (IVW: OR = 1.069 [95% CI: 1.035–1.104, *p* < 0.001]; MR‐Egger: *p* = 0.010; weighted median: *p* = 0.013; weighted mode: *p* = 0.011) showed a significant positive association with HF. Beta‐citrylglutamate (BCG) also displayed a heightened risk (IVW: OR = 1.071 [95% CI: 1.022–1.121, *p* = 0.011]). Additionally, N‐acetylglucosamine/N‐acetylgalactosamine (GlcNAc/GalNAc) (IVW: OR = 1.047 [95% CI: 1.013–1.082, *p* = 0.006]; weighted median: *p* = 0.022), X‐24801 (IVW: OR = 1.060 [95% CI: 1.021–1.102, *p* = 0.040]). Similarly, X‐21283 had an OR of 1.028 [95% CI: 1.007–1.050, *p* = 0.009], and X‐22776 exhibited an OR of 1.107 [95% CI: 1.027–1.193, *p* = 0.008] in the IVW method, both suggesting increased susceptibility to HF. The MR‐Egger method supports the IVW method result of 2PY and X‐24801; the weighted median method supports the result of 2PY, GlcNAc/GalNAc, and X‐24801; the weighted mode supports the result of 2PY.

On the other hand, six metabolites exhibited a protective effect against HF, with OR values less than 1. Palmitoylcarnitine showed a significant protective effect (IVW: OR = 0.941 [IVW: 95% CI: 0.902–0.981, *p* = 0.004]), as did 3‐hydroxylaurate (IVW: OR = 0.904 [95% CI: 0.839–0.974, *p* = 0.008]). 2 − butenoylglycine levels were similarly associated with a lower risk of HF (IVW: OR = 0.937 [95% CI: 0.901–0.974, *p* < 0.001]), along with X‐23659, which also showed a protective association (IVW: OR = 0.956 [95% CI: 0.926–0.987, *p* = 0.006]). X‐25422 exhibited an OR of 0.940 (95% CI: 0.900–0.980, *p* = 0.004), and X‐24546 exhibited an OR of 0.932 (95% CI: 0.897–0.969, *p* = 0.004) in IVW method, both suggesting decreased susceptibility to HF. The weighted median method supports the IVW method result of X‐23659 and X‐24546; simple mode method supports the result of X‐24546; weighted mode supports the result of X‐23659.

Tests for bias (Egger regression) and heterogeneity (Cochran′s *Q*) showed no significant issues, and a leave‐one‐out analysis confirmed the stability of our findings (Table S9). These results support the validity of the IVs and the causal relationship between exposure and outcome.

### 3.3. Mediation Roles of uPA on HF via X‐22776

**Figure 5:**
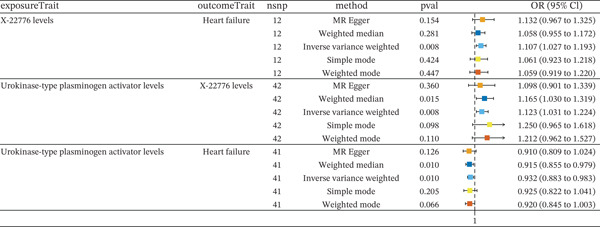
Forest plot depicting the association between uPA and X‐22776 with HF.

Previously, we identified 8 inflammatory cytokines that have a unidirectional effect on HF, as well as 12 metabolites that may influence HF. Now, we are exploring the causal relationships between these inflammatory cytokines and metabolites. Figure 5 demonstrates the associations between various inflammatory cytokine levels and metabolites with the risk of HF, as analyzed through different MR methods.

The analysis identified a significant association between X‐22776 levels and HF using the IVW method (OR = 1.107, 95% CI [1.027 to 1.193], *p* = 0.008). Other methods, such as weighted median, MR‐Egger, simple mode, and weighted mode, showed no significant associations, suggesting some variability depending on the method used. uPA levels were significantly associated with both X‐22776 levels (OR = 1.123, 95% CI [1.031 to 1.224], *p* = 0.008) and HF (OR = 0.932, 95% CI [0.883 to 0.983], *p* = 0.010) in the IVW method. The weighted median method further supported the association with X‐22776 levels (OR = 1.165, 95% CI [1.030 to 1.319], *p* = 0.015) and HF (OR = 0.915, 95% CI [0.855 to 0.979], *p* = 0.010).

Table 1 summarizes the mediation analysis of uPA on HF risk via X‐22776. The total effect of uPA on HF (*β*_all) was −0.0704 (95% CI: −0.1247 to −0.0161). The effect of uPA on the mediator X‐22776 (*β*_1_) was 0.1162 (95% CI: 0.0305 to 0.2020), and the effect of X‐22776 on HF (*β*_2_) was 0.1020 (95% CI: 0.0271 to 0.1769). The indirect effect was 0.0119 (95% CI: 0.0007 to 0.0224), with a mediated proportion of −16.8% (95% CI: −34.7% to 1.0%). The negative mediated proportion reflects that the indirect effect through X‐22776 opposes the direct protective effect of uPA, suggesting a complex interplay between uPA and this metabolite in HF pathogenesis.

**Table 1:** Mediation effect of uPA on HF via X‐22776.

beta.1	beta.2	beta.all	Mediated effect	Mediated proportion (%)
0.1162	0.1020	−0.0704	0.0119	−16.8%
(0.0305, 0.2020)	(0.0271, 0.1769)	(−0.1242, −0.0167)	(0.0007, 0.0244)	(1%, −34.7%)

*Note:* beta1: The causal role of inflammatory cytokines on metabolites. beta2: The causal role of metabolites on HF. beta.all: The causal role of inflammatory cytokines on HF. *β* (mediated effect) = *β*(beta1)∗*β*(beta2). The mediated proportion = *β* (mediated effect)/(beta all).

### 3.4. Pathway Enrichment Analysis

**Figure 6:**
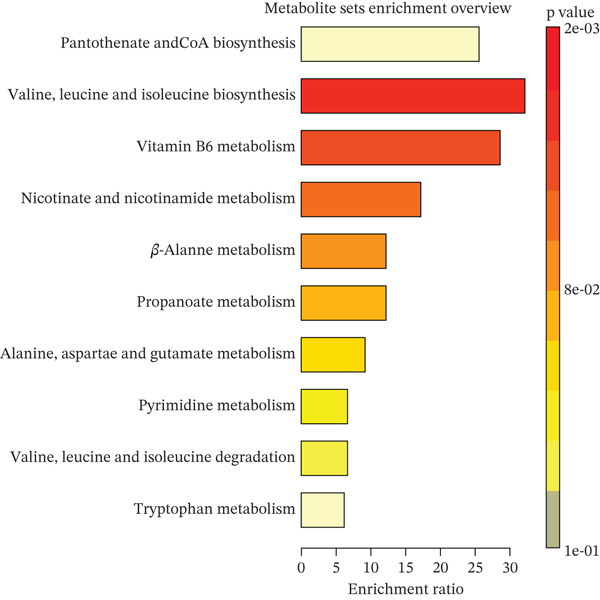
The metabolite sets enrichment analysis of HF.

We performed pathway and enrichment analyses on 53 metabolites significantly associated with HF in the discovery dataset. Detailed information on these important known metabolites is provided in Table S10. Using the RaMP database, we identified 10 significant metabolic pathways enriched in HF (Figure 6). These pathways encompass a wide range of metabolic processes, including amino acids, vitamins, and carbohydrates. Notable enriched pathways include those related to valine, alanine, aspartate, and glutamate, reflecting the synthesis and degradation of amino acids. Additionally, some nucleic acid metabolism pathways (e.g., pyrimidine) were identified, highlighting the importance of nucleic acid metabolites in physiological processes.

## 4. Discussion

Our study provides genetic evidence supporting that both inflammatory cytokines and metabolites play critical roles in the development and progression of HF. Specifically, our findings suggest that certain inflammatory cytokines may confer protective effects, whereas others are associated with an increased risk of HF.

MMPs are a class of enzymes capable of degrading structural matrix proteins, and their elevated levels or activity are often associated with cardiac diseases [25]. MMP‐1 is primarily known for its role in extracellular matrix (ECM) remodeling. In the context of HF, MMP‐1 contributes to adverse cardiac remodeling by degrading ECM components, leading to myocardial fibrosis and ventricular dilation [26]. Although there have been no direct studies on MMP‐1 in HF, elevated levels of MMP‐1 have been observed in patients with acute myocardial infarction, correlating with disease severity and poorer outcomes [27]. Consequently, targeting MMP‐1 to modulate ECM remodeling has been proposed as a potential therapeutic strategy to prevent the progression of HF [28]. Previous studies have primarily focused on the effects of TNF‐*α* on heart function, with limited attention given to TNF‐*β* [29–31]. Both TNF‐*α* and TNF‐*β* belong to the TNF family and are related cytokines that have overlapping but distinct roles in immune regulation and inflammation. TNF generally promotes myocardial inflammation, apoptosis, and fibrosis, contributing to adverse cardiac remodeling and systolic dysfunction [32]. Although TNF‐*β* has been less studied in clinical settings, it is recognized for its role in immune regulation and certain autoimmune conditions [33, 34]. Our study did not find evidence of a direct effect of TNF‐*α* on HF but found that TNF‐*β* is associated with an increased risk of HF. Previous studies have suggested that FGF15/19 helps regulate lipid and glucose metabolism, working alongside adiponectin to prevent atherosclerosis and other metabolic diseases [35, 36]. Additionally, FGF19 has been reported to protect against the deleterious effects of diabetic cardiomyopathy, atherosclerosis, and coronary artery disease (CAD) [37–39]. Reduced serum levels of FGF19 and adiponectin in CAD patients have been associated with increased disease severity, suggesting that FGF19 could serve as an inverse serum biomarker of CAD [40]. However, our study found the opposite, indicating that elevated FGF19 levels are detrimental to heart function. This elevation has been linked to cardiac dysfunction and adverse remodeling in HF, potentially due to its impact on lipid metabolism and the promotion of cardiac steatosis [41].

Although direct evidence linking DNER to HF is limited, researchers found peak values of DNER were reduced in patients with ST‐segment elevation myocardial infarction treated with therapeutic hypothermia, suggesting that DNER may play a regulatory role in maintaining cardiac structure and function [42]. Although we have found the potential beneficial effect of DNER on cardiac function, the mechanism of its research remains to be further studied. IL‐10 is a mononuclear cell product that potently inhibits the production of proinflammatory cytokines (such as IL‐1, IL‐6, and TNF‐*α*), and also regulates ECM metabolism and angiogenesis [43]. In the context of HF, IL‐10 has been shown to exert cardioprotective effects by inhibiting inflammatory responses and oxidative stress [44]. Clinical studies have suggested that IL‐10 was identified as an independent predictor of HF readmission [45]. TNFSF12, also known as TWEAK, a member of the tumor necrosis factor ligand superfamily, is a cytokine involved in inflammation, cell survival, and tissue remodeling [46]. TNFSF12 has been shown to exert both protective and detrimental effects on the cardiovascular system, depending on the context [47]. In HF, TNFSF12 may also play a role in tissue repair and angiogenesis [48, 49]. LIFR has been recognized as a protective factor in HF. LIF is a highly effective remodeling factor for adult cardiomyocytes. Under ischemic conditions, LIF exhibits cardioprotective effects by responding to oxidative stress and preventing cell death [50]. LIF/LIFR/gp130 axis can inhibit cardiac fibrosis and hypertrophy, promoting cardiomyocyte survival and enhancing cardiac function [51, 52].

As an ECM modulator, uPA mediates the degradation of fibrin and other ECM proteins, playing a vital role in maintaining cardiac structure and function. Elevated uPA levels have been shown to reduce cardiac fibrosis, improve left ventricular function, and facilitate tissue repair following myocardial injury [53]. These protective effects of uPA could potentially be attributed to its involvement in fibrinolysis, preventing excessive ECM accumulation, which is a key factor in the development of myocardial fibrosis and HF progression [54]. Moreover, our study identified a significant association between the metabolite X‐22776 and HF risk, with uPA likely influencing HF through its impact on this metabolite. X‐22776, as a new metabolite, has been discovered in the human body, and its functional effects remain to be further studied. By modulating metabolites such as X‐22776, uPA could indirectly influence metabolic processes, further underscoring its importance in HF pathophysiology.

Our MR analysis identified several metabolites, including 2PY, BCG, GlcNAc/GalNAc, X‐24801, X‐21283, and X‐22776, as being associated with an increased risk of HF, suggesting their potential involvement in disease pathogenesis through various mechanisms. Conversely, metabolites such as palmitoylcarnitine, 3‐hydroxylaurate, 2‐butenoylglycine, X‐23659, X‐25422, and X‐24546 were associated with a lower HF risk, indicating potential protective roles. Currently, research on metabolites is still in its early stages; among them, 2PY and palmitoylcarnitine are the metabolites that have been relatively well‐studied. 2PY is a metabolite of nicotinamide adenine dinucleotide (NAD), involved in oxidative stress and energy metabolism [55]. Elevated 2PY levels have been linked to mitochondrial dysfunction, a hallmark of HF [56]. Mitochondria are crucial for maintaining cardiac energy supply, and impairments in their function can lead to an energy deficit in cardiomyocytes, contributing to HF [57]. Palmitoylcarnitine is a long‐chain acylcarnitine involved in fatty acid transport and mitochondrial *β*–oxidation, processes that are crucial for cardiac energy metabolism. In a healthy heart, fatty acids are the primary source of energy, with palmitoylcarnitine serving as an essential carrier for the transport of fatty acids into the mitochondria [58]. Elevated levels of palmitoylcarnitine are associated with increasing mitochondrial activity, contributing to energy depletion in cardiac cells, which may be instrumental in myocardial function [59, 60]. The functions of the metabolites BCG, GlcNAc/GalNAc, X‐24801, X‐21283, X‐22776, 2‐butenoylglycine, 3‐hydroxylaurate, X‐23659, X‐25422, and X‐24546 remain to be further explored, even though we have identified their association with HF.

Despite the robustness of our MR analysis, several limitations must be acknowledged. First, MR relies on genetic variants as IVs, which, although reducing confounding, cannot account for all sources of bias, such as pleiotropy or gene‐environment interactions. Although we employed sensitivity analyses to address pleiotropy, residual bias cannot be completely excluded. Second, regarding the selection of IVs, we adopted a *p* value threshold of *p* < 1 × 10^−5^, which is less stringent than the conventional genome‐wide significance level (*p* < 5 × 10^−8^). This relaxed threshold was necessary because applying stricter criteria would have resulted in an insufficient number of SNPs to serve as IVs for cytokines and metabolites. However, this less stringent threshold may introduce weak instrument bias, potentially affecting the precision of causal estimates. To mitigate this concern, we calculated *F*‐statistics to ensure instrument strength and performed multiple sensitivity analyses to assess the robustness of our findings. Third, our study is limited by the availability of GWAS data, and the metabolites and cytokines included in our analysis may not represent the entire spectrum of biological pathways involved in HF. Future research should incorporate more diverse datasets to validate these findings across different populations. Lastly, although this study identifies causal relationships, further experimental validation is warranted to clarify the underlying biological mechanisms. Future studies should consider experimental validation in animal models of HF to confirm the causal roles of the identified cytokines and metabolites, particularly uPA and X‐22776. Metabolomic profiling in HF subtypes, such as HF with preserved ejection fraction (HFpEF) versus HF with reduced ejection fraction (HFrEF), may reveal distinct metabolic signatures and pathway involvement. Additionally, functional characterization of the uncharacterized metabolites identified in this study (X‐24801, X‐21283, X‐22776, X‐23659, X‐25422, and X‐24546) using mass spectrometry–based structural elucidation and in vitro cellular assays would be valuable to understand their biological roles in cardiac pathophysiology.

## 5. Conclusion

In conclusion, this MR study provides important insights into the causal roles of inflammatory cytokines and metabolites in the development of HF. These findings underscore the complex interaction between inflammation, metabolism, and HF, offering potential targets for therapeutic intervention. Future research should focus on elucidating the underlying biological mechanisms and validating these findings in diverse populations.

## Author Contributions

Yanbing Yao conceived and designed the study, performed data analysis, and interpreted the results, contributing to drafting and revising the manuscript critically for important intellectual content. Linghui Tang assisted in the study design and data analysis, significantly contributing to the interpretation of results and the manuscript′s drafting and revision. Feng Huang provided critical input on the study design and methodology, supervised the overall project, ensured data integrity, and contributed to the manuscript preparation and revision. Zhiyu Zeng coordinated the research process, provided expert guidance in data interpretation, supervised the entire study, secured funding, and revised the manuscript critically for important intellectual content. Yanbing Yao and Linghui Tang contributed equally to this work, whereas Feng Huang and Zhiyu Zeng are the corresponding authors.

## Funding

This study was supported by the Guangxi Key Laboratory of Precision Medicine in Cardio‐Cerebrovascular Diseases Control and Prevention (22‐035‐18), The Central Government Guides Local Science and Technology Development Funding Projects (Guike ZY23055038), National Natural Science Foundation of China (82460075), Guangxi Science and technology planning project (Guike AB24010178), and Guangxi Clinical Research Center for Cardio‐cerebrovascular Diseases (AD17129014).

## Disclosure

All authors have read the manuscript and agreed to its publication.

## Ethics Statement

This study relied on publicly accessible summary statistics from previously published research and consortia. Each original study involved in this research had received ethical clearance from their respective review boards, and all participants had given informed consent. Notably, this research did not involve the use of individual‐level data, thereby eliminating the need for new ethical review board approval.

## Conflicts of Interest

The authors declare no conflicts of interest.

## Supporting Information

Additional supporting information can be found online in the Supporting Information section.

## Supporting information


**Supporting Information 1** Figure S1: MR leave‐one‐out sensitivity analysis for inflammatory cytokines on heart failure.

**Supporting Information 2** Figure S2: MR leave‐one‐out sensitivity analysis for metabolites on heart failure.

**Supporting Information 3** Table S1: IVs for 1400 metabolites.

**Supporting Information 4** Table S2: IVs for 91 inflammatory cytokines.

**Supporting Information 5** Table S3: IVs for heart failure.

**Supporting Information 6** Table S4: Heterogeneity analysis of inflammatory cytokines on heart failure.

**Supporting Information 7** Table S5: Pleiotropy analysis of inflammatory cytokines on heart failure.

**Supporting Information 8** Table S6: OR of reverse MR analysis.

**Supporting Information 9** Table S7: Sensitive analysis of reverse MR.

**Supporting Information 10** Table S8: Twelve metabolites.

**Supporting Information 11** Table S9: Sensitive analysis of metabolites on heart failure.

**Supporting Information 12** Table S10: Metabolites enrichment analysis.

## Data Availability

The data that supports the findings of this study are available in the Supporting Information of this article.
