# Supplementary figures and images for: Genetic Evidence Linking Inflammatory Cytokines and Blood Metabolites to Heart Failure Risk
Source: Cardiovasc Ther. 2026 May 11;2026:6340671. doi: 10.1155/cdr/6340671 (PMC13159094; doi:10.1155/cdr/6340671)

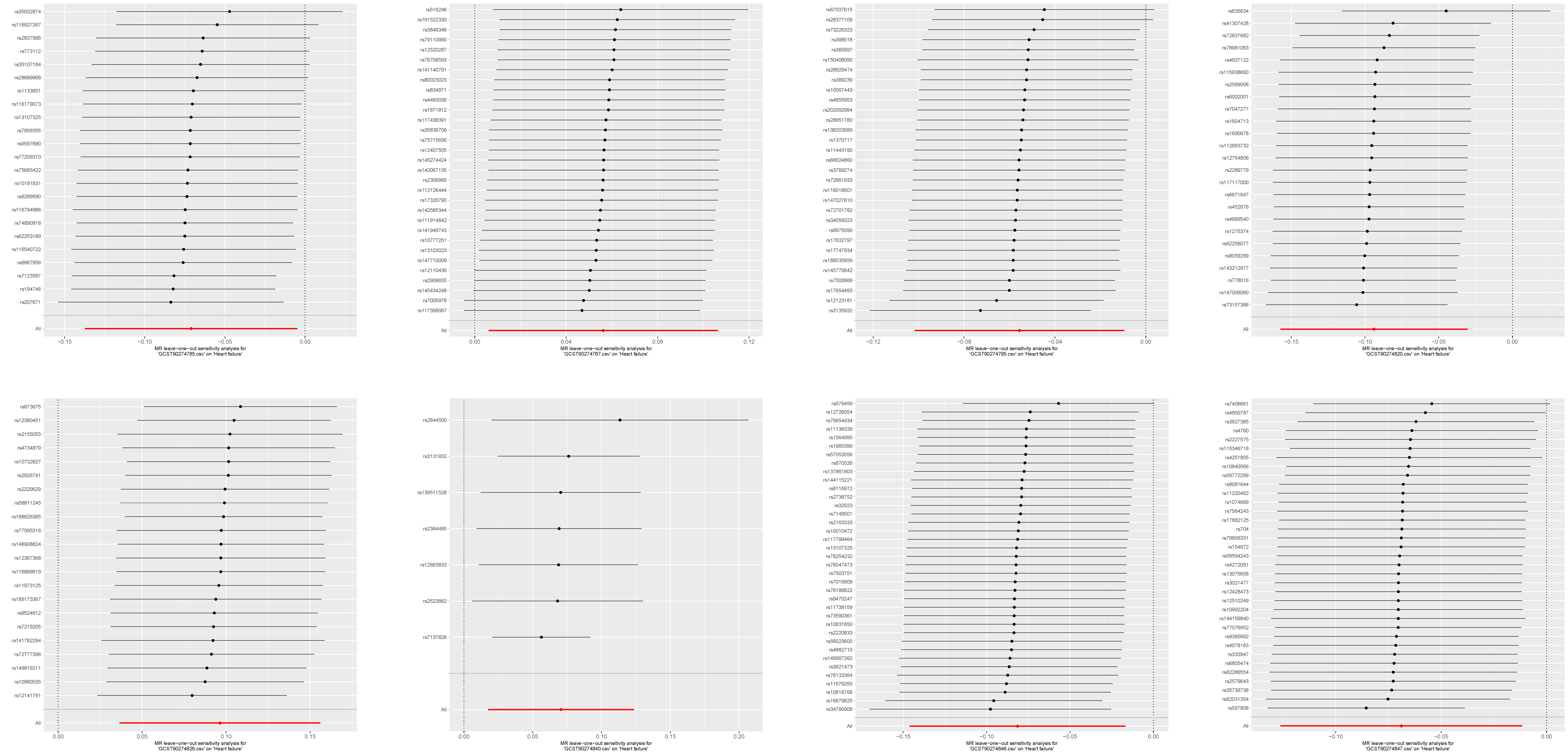

Supplement: Supplementary file 1 — Supporting Information 1 Figure S1: MR leave‐one‐out sensitivity analysis for inflammatory cytokines on heart failure. [file CDR-2026-6340671-s003.tif]

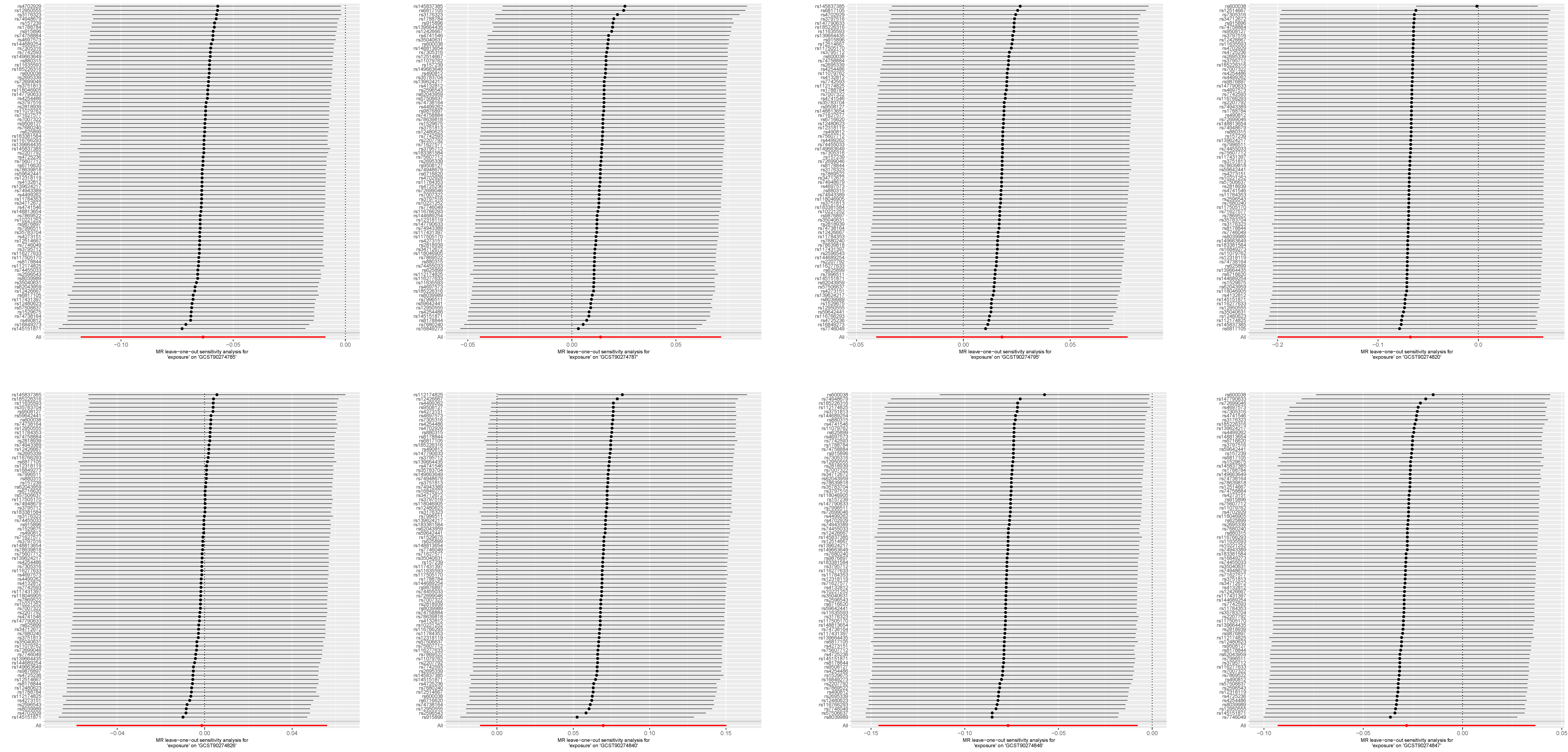

Supplement: Supplementary file 2 — Supporting Information 2 Figure S2: MR leave‐one‐out sensitivity analysis for metabolites on heart failure. [file CDR-2026-6340671-s004.tif]
